# The Technology-Oriented Pathway for Auxiliary Diagnosis in the Digital Health Age: A Self-Adaptive Disease Prediction Model

**DOI:** 10.3390/ijerph191912509

**Published:** 2022-09-30

**Authors:** Zhiyuan Hao, Jie Ma, Wenjing Sun

**Affiliations:** 1 School of Business and Management, Jilin University, Changchun 130012, China; 2 Information Resource Research Center, Jilin University, Changchun 130012, China

**Keywords:** machine learning, disease prediction model, auxiliary diagnosis, digital health, medical informatics

## Abstract

The advent of the digital age has accelerated the transformation and upgrading of the traditional medical diagnosis pattern. With the rise of the concept of digital health, the emerging information technologies, such as machine learning (ML) and data mining (DM), have been extensively applied in the medical and health field, where the construction of disease prediction models is an especially effective method to realize auxiliary medical diagnosis. However, the existing related studies mostly focus on the prediction analysis for a certain disease, using models with which it might be challenging to predict other diseases effectively. To address the issues existing in the aforementioned studies, this paper constructs four novel strategies to achieve a self-adaptive disease prediction process, i.e., the hunger-state foraging strategy of producers (PHFS), the parallel strategy for exploration and exploitation (EEPS), the perturbation–exploration strategy (PES), and the parameter self-adaptive strategy (PSAS), and eventually proposes a self-adaptive disease prediction model with applied universality, strong generalization ability, and strong robustness, i.e., multi-strategies optimization-based kernel extreme learning machine (MsO-KELM). Meanwhile, this paper selects six different real-world disease datasets as the experimental samples, which include the Breast Cancer dataset (cancer), the Parkinson dataset (Parkinson’s disease), the Autistic Spectrum Disorder Screening Data for Children dataset (Autism Spectrum Disorder), the Heart Disease dataset (heart disease), the Cleveland dataset (heart disease), and the Bupa dataset (liver disease). In terms of the prediction accuracy, the proposed MsO-KELM can obtain ACC values in analyzing these six diseases of 94.124%, 84.167%, 91.079%, 72.222%, 70.184%, and 70.476%, respectively. These ACC values have all been increased by nearly 2–7% compared with those obtained by the other models mentioned in this paper. This study deepens the connection between information technology and medical health by exploring the self-adaptive disease prediction model, which is an intuitive representation of digital health and could provide a scientific and reliable diagnostic basis for medical workers.

## 1. Introduction

With the rapid development of the economy and technology, public demands for improving healthcare are getting stronger and how to utilize information technology to achieve auxiliary diagnosis has received increasing social attention [1,2,3,4]. Moreover, the spread of the concept of digital health [5,6,7] promotes deep integration between information technology and healthcare, where a large number of machine learning (ML) models and data mining (DM) methods have been introduced into the traditional medical diagnosis pattern. To date, there are various existing studies adopting the technologies of ML and DM to predict diseases, such as predicting stable MCI patients [8], forecasting nuanced yet significant MT errors of clinical symptoms [9], survival risk prediction for esophageal cancer [10], conducting breast cancer diagnosis [11], preconception prediction for gestational diabetes mellitus [12], predicting Alzheimer’s disease [13], and heart disease prediction [14,15].

Disease prediction analysis based on ML and DM is a research trend in medical informatics. These research findings could provide a scientific and reliable diagnostic basis for medical workers and provide an effective technical support for the early intervention of related diseases. For example, Derevitskii et al. construct a hybrid predictive modelling for Thyrotoxic atrial fibrillation, which could be used as part of a decision support system for medical staff who work with thyrotoxicosis patients [16]. Similarly, Muhammad et al. develop a machine leaning predictive model for coronary artery disease (CAD), which could be used to develop an expert system for diagnosis of CAD patients in Nigeria [17]. Nevertheless, these aforementioned studies still contain a critical limitation, i.e., the existing proposed models mainly focus on a certain disease and mostly neglect expanding the application’s universality for predicting various other diseases, which means that these models may not obtain accurate prediction results in analyzing those other diseases. Furthermore, a model with better performance when predicting different diseases would be more significant for the medical workers and the auxiliary diagnosis process. Therefore, constructing a disease prediction model with self-adaptive ability, strong generalization ability, and strong robustness is the motivation to explore the technology-oriented pathway for auxiliary diagnosis in digital health age.

There may be large differences among the disease data from the real world, such as different attribute dimensions and different inner structures. In fact, an ideal disease prediction model which meets current medical needs should combine prediction accuracy with broad applicability. To this end, we take the kernel extreme learning machine (KELM) as the base model, which has advantages in generalization ability and robustness, and we introduce an improved swarm intelligence optimization algorithm to optimize the KELM, i.e., sparrow search algorithm (SSA) with the enhanced global searching ability (EGSSA). Finally, we design a novel disease prediction model, i.e., multi-strategies optimization-based kernel extreme learning machine (MsO-KELM). The main contributions and innovations of the MsO-KELM are highlighted as follows:

(1) To effectively predict various diseases, we utilize the EGSSA to optimize the base model by designing four novel strategies, i.e., the hunger-state foraging strategy of producers (PHFS), the parallel strategy for exploration and exploitation (EEPS), the perturbation–exploration strategy (PES), and the parameter self-adaptive strategy (PSAS). These strategies can enhance the prediction accuracy of the model and allow the model to be applied to various diseases.

(2) To verify the prediction performance of the proposed MsO-KELM, we adopt six different disease datasets as the experimental samples, consisting of the Breast Cancer dataset (cancer), the Parkinson dataset (Parkinson’s disease), the Autistic Spectrum Disorder Screening Data for Children dataset (Autism Spectrum Disorder), the Heart Disease dataset (heart disease), the Cleveland dataset (heart disease), and the Bupa dataset (liver disease), and we evaluate the prediction performance by four different evaluation metrics, i.e., the ACC, the sensitivity, the specificity, and the MCC. Notably, the ACC is the most significant metric to evaluate the prediction accuracy.

(3) To elaborate the details of the MsO-KELM, we conduct two-stage experiments in this paper. The first experiment is mainly to prove the better optimization performance of the EGSSA, which is the basis for achieving the self-adaptive characteristic of the MsO-KELM. The second experiment is mainly to compare the MsO-KELM with other state-of-the-art prediction models.

**Figure 1:**
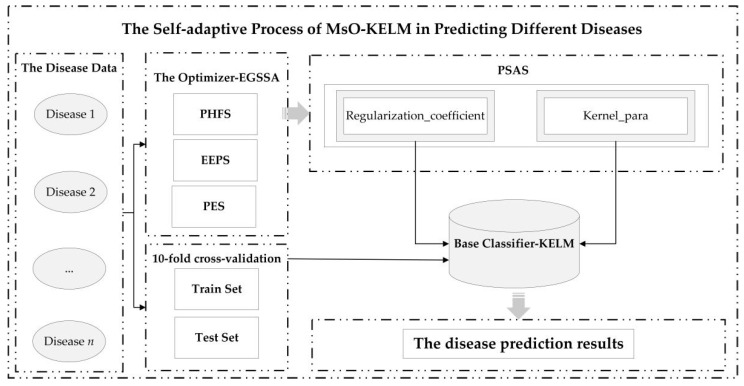
The self-adaptive process of the MsO-KELM in predicting different diseases.

The self-adaptive prediction process of the MsO-KELM in analyzing the different diseases is shown in Figure 1.

## 2. Preparation

### 2.1. Disease Data Description

In this paper, we select six different real-world disease datasets as the experiment samples (these data are available at https://archive.ics.uci.edu/mL/index.php, accessed on 6 June 2022), i.e., the Breast Cancer dataset (cancer), the Parkinson dataset (Parkinson’s disease), the Autistic Spectrum Disorder Screening Data for Children dataset (Autism Spectrum Disorder), the Heart Disease dataset (heart disease), the Cleveland dataset (heart disease), and the Bupa dataset (liver disease). The reasons for selecting these six disease datasets are shown as follows:

- They are the common diseases in the real world;
- These disease data are extensively utilized by numerous investigators;
- These disease data have different internal structures and different diagnosis indicators.

The characteristics of these six disease datasets are shown in Table 1.

### 2.2. The Base Disease Classifier—Kernel Extreme Learning Machine

The KELM is an excellent classifier which has advantages in generalization ability and learning speed [18,19,20,21,22]. Since its emergence, the KELM has been extensively studied by numerous investigators for problems such as hyperspectral image classification [23], data classification in enterprise cloud data [24], time-varying distributed parameter systems [25], and intrusion detection [26]. Notably, there are two significant parameters in the original KELM, i.e., the *k* value (kernel parameter) and the *c* value (regularization coefficient), which easily fall into local optimum in the original searching process [27,28] and can influence the final prediction accuracy. The main calculation processes of KELM are shown as follows:

(1) $$\mathrm{Objective}\_\mathrm{function}\_\mathrm{ELM}=hid\left(x\right)\times H\_outp^{T}\times {\left(\frac{I\_matrix}{Coefficient\_r}+H\_outp\times H\_outp^{T}\right)}^{-1}\times L$$

(2) $$\ker\mathrm{nel}\_\mathrm{matrix}=H\_\mathrm{outp}\times {H\_\mathrm{outp}}^{T}=\mathrm{hid}\left(x_{i}\right)\times \mathrm{hid}\left(x_{j}\right)=k\left(x_{i},\;x_{j}\right)$$

(3) $$\mathrm{Objective}\_\mathrm{function}\_\mathrm{KELM}={\left[\begin{array}{l} k\left(x,\;x_{1}\right)\\ \vdots \\ k\left(x,\;x_{n}\right) \end{array}\right]}^{T}\times {\left(\frac{I\_matrix}{Coefficient\_r}+\ker nel\_matrix\right)}^{-1}\times L$$

where the *Objective_function_ELM* and *Objective_function_KELM* indicate the learning objective function of ELM and that of KELM, respectively. The *hid*(*x*) and *H_outp* represent the feature mapping matrix of hidden layer [18], *I_matrix* indicates the identity matrix, *Cofficient_r* indicates the regularization coefficient, *L* indicates the expectation matrix, and *k*(*x_i_*, *x_j_*) indicates the kernel function. In this paper, we adopt the Gaussian kernel function, and the kernel parameter *k* indicates the kernel width.

### 2.3. The Base Optimization Tool—Sparrow Search Algorithm

In order to improve the prediction performance of the base-classifier, we take SSA as a base-optimizer and conduct the improvements on SSA to design a more effective optimizer, i.e., the EGSSA. As a recent meta-heuristic algorithm [29], SSA has been applied in many real-world problems [30,31,32,33,34] because of the advantages in convergence speed and exploitation ability. There are two significant population roles in the optimization process of SSA, i.e., producers and scroungers, in which the producers could be regarded as the leader with a higher fitness [30]. The location update processes of these two roles are shown as follows:

(4) $${\mathrm{posi}}_{i,\;d}^{t+1}=\left\{ \begin{array}{l} posi_{i,\;d}^{t}\cdot \exp\left(\frac{-i}{\alpha \cdot iter_{\max}}\right),\;R\;<\;ST\\ posi_{i,\text{ʠ}d}^{t}+R\mathrm{andom}\cdot Matrix,\;R\;\geq \;ST \end{array}\right.$$

(5) $${\mathrm{posi}}_{i,\;d}^{t+1}=\left\{ \begin{array}{l} Random\cdot \exp\left(\frac{posi_{worst}-posi_{i,\;d}^{t}}{i^{2}}\right),\;i\;>\;n/2\\ posi_{a}^{t+1}+\left|posi_{i,\;d}^{t}-posi_{a}^{t+1}\right|\cdot V^{+}\cdot Matrix,\;i\;\leq \;n/2 \end{array}\right.$$

(6) $${\mathrm{posi}}_{i,\;d}^{t+1}=\left\{ \begin{array}{l} posi_{\mathrm{best}}^{t}+\beta \cdot \left|{\mathrm{posi}}_{i,\;d}^{t}-posi_{\mathrm{best}}^{t}\right|\;fitness_{i}\;>\;fitness_{g}\\ {\mathrm{posi}}_{i,\;d}^{t}+\psi \;\left(\frac{\left|{\mathrm{posi}}_{i,\;d}^{t}-posi_{\mathrm{worst}}^{t}\right|}{\left(fitness_{i}-fitness_{w}\right)+\xi }\right)\;fitness_{i}\;=\;fitness_{g} \end{array}\right.$$

Equation (4) describes the location update process of producer, the ${\mathrm{posi}}_{i,\;d}^{t+1}$ indicates the current location of the *i^th^* sparrow individual in the *d^th^* dimensional space when the populations are carrying out the *t^th^* iteration [30], *t* and *i* indicate the current iterations and current sparrow individual, respectively. The *α*, *R*, and *Random* are the random parameters set manually, the *ST* is a warning threshold, the values of which could be set in (0.5, 1) [30]. The *Matrix* indicates a row vector where each element value is set to 1 and the dimensions are *d* [30]. Equation (5) describes the location update process of scrounger, the ${posi}_{a}^{t+1}$ indicates the optimal location searched by producer, the *posi_worst_* indicates the worst location in the current iteration [30], the *n* is the sparrow population size, and the *V* indicates a row vector where each element value is set to 1 or −1 randomly and the dimensions are *d*. Equation (6) describes the location update process of detection sparrow, the ${posi}_{best}^{t}$ indicates the optimal location when the populations are carrying out the *t^th^* iteration, and the *β*, *ψ*, and *ξ* indicate adjustment parameters [30].

### 2.4. The Introduction of Evaluation Metrics

To evaluate the disease prediction performance of the proposed MsO-KELM, we adopt the four evaluation metrics in this paper, i.e., classification accuracy (ACC, the value range is from 0 to 1.0) [18], sensitivity (the value range is from 0 to 1.0) [18], specificity (the value range is from 0 to 1.0) [18], and Mathews correlation coefficient (MCC, the value range is from −1.0 to 1.0) [18]. The ACC emphasizes the number of samples which are correctly predicted (the most significant evaluation metric to measure the classification performance of a model), the sensitivity shows the ability to correctly predict a positive sample among all positive samples, the specificity indicates the ability to correctly predict a negative sample among all negative samples, and the MCC mainly shows the reliability of a model (the closer the MCC value is to 1, the more accurate and effective the model is). The calculation processes of these four evaluation metrics are shown as follows:

(7) $$ACC=\frac{TP+TN}{TP+TN+FP+FN}$$

(8) $$sensitivity=\frac{TP}{TP+FN}$$

(9) $$specificity=\frac{TN}{TN+FP}$$

(10) $$MCC=\frac{TP\times TN-FP\times FN}{\sqrt{\left(TN+FP\right)\times \left(TN+FN\right)\times \left(TP+FN\right)\times \left(TP+FP\right)}}$$

where the *TP* indicates the number of samples with the positive prediction result and the positive label, the *TN* indicates that the number of samples with the negative prediction result and the negative label, the *FP* indicates that the number of samples with the positive prediction result and the negative label, and the *FN* indicates that the number of samples with the negative prediction result and the positive label.

## 3. The Proposed Methodology

The core idea of the proposed methodology is to construct a self-adaptive disease prediction model with high accuracy, strong generalization ability, and strong robustness. Therefore, we select an excellent base-classifier (KELM) and design an enhanced meta-heuristic algorithm (EGSSA) as the optimizer to finally construct the self-adaptive disease prediction model, i.e., the MsO-KELM. Specifically, there are four novel strategies in the MsO-KELM, i.e., the hunger-state foraging strategy of producers (PHFS), the parallel strategy for exploration and exploitation (EEPS), the perturbation–exploration strategy (PES), and the parameter self-adaptive strategy (PSAS), where the EGSSA consists of the PHFS, the EEPS, and the PES. In addition, the PSAS will act on a parameter acquisition mechanism which is formed by combining the EGSSA with the KELM. In this section, the technological details of the MsO-KELM will be discussed as follows:

### 3.1. Foraging Strategy of Producers in Hunger-State (PHFS)

In the original SSA, the producers could be regarded as the leader roles in the sparrow populations, which are responsible for searching for food-rich positions and providing foraging directions for all scroungers, and the scroungers could follow the producers to achieve the foraging process. In that case, if the producers could expand the searching range to find a safer and more adequate position, it would provide more possibilities for scroungers to improve the foraging rate and finally enhance the global convergence performance.

However, the existing location update mechanism mainly focuses on the exploitation ability (local searching ability) of the original SSA, which may cause the producers trapping into the local optimum situation. To address this issue and enhance the exploration ability (global searching ability), we introduce the hunger games search algorithm (HGS) [35,36] into the original SSA to construct the PHFS for expanding the searching range of optimal position. Specifically, the PHFS is a hybrid strategy, which retains the advantage in exploitation ability of the original SSA while combining the exploration approach of the HGS algorithm with the location update mechanism of the producers.

In the original SSA, the location update process of producers is shown as Equation (4), and the key calculation function affecting the convergence efficiency is shown in Equation (11):

(11) $$\mathrm{function}\left(i\right)=\exp\left(\frac{-i}{\alpha \cdot iter_{\max}}\right)$$

where the *α* and *iter_max_* are the parameters set manually. It is clearly shown that Equation (11) has a descending trend and will eventually converge to 0, which means the producers can easily repeat the searching behavior at a certain position with the number of individuals increasing, and eventually fall into a local optimum. By contrast, the HGS has a significant advantage in exploration ability, and the hungry feature function affecting the convergence efficiency is shown in Equation (12):

(12) function(j)=1−exp(−|par_manual−j|)

where the *par_maunal* is a parameter set manually. It is clearly shown that Equation (12) is not a single ascending or descending trend, but the function trend is affected by the parameter value. When the inputting value is less than the *par_maunal* value, the function could have an ascending trend with the inputting value increasing. Moreover, when the inputting value is larger than the *par_maunal* value, the function could have a descending trend with the inputting value increasing. Therefore, introducing the hungry feature of HGS into the searching behavior of producers could expand the searching range of optimal position, and ultimately enhance the exploration ability of SSA.

**Figure 2:**
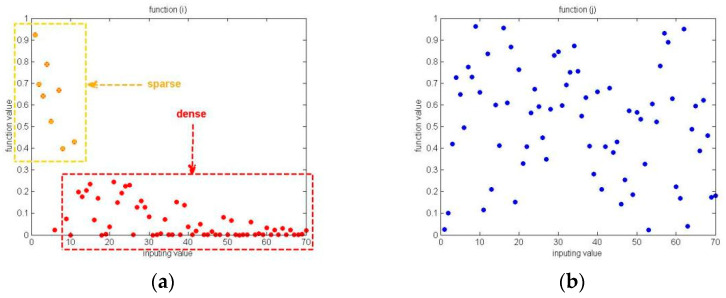
The searching result figures based on Equations (11) and (12). (**a**) searching processes based on Equation (11); (**b**) searching processes based on Equation (12).

The searching processes based on Equations (11) and (12) are shown in Figure 2.

According to Figure 2, we could find that the original location update method can easily search in a local space (like the red region in Figure 2a), but the hungry roles of HGS could search in a relatively global region (like Figure 2b). Therefore, the PHFS could be described as follows:

(13) $${\mathrm{posi}\_\mathrm{new}}_{i,\;d}^{t+1}=\left(1-\exp\left(-\left|posi\_current_{best}-posi\_current_{i,\;d}^{t}\right|\right)\right)\cdot \mathrm{rand}\cdot ο$$

where the meaning of ${\mathrm{posi}\_\mathrm{new}}_{i,\;d}^{t+1}$ is similar to that of ${\mathrm{posi}}_{i,\;d}^{t+1}$ in Equation (4), the ${posi\_current}_{i,d}^{t}$ indicates the location of last iteration, the *rand* is a random number, and the *o* is an adjustment parameter which is set to 2 in this paper.

### 3.2. Parallel Strategy for Exploration and Exploitation (EEPS)

As shown in Equation (4), we could find that the location update process of producers consists of two different stages. The PHFS acts on stage 1 (*R* < *ST*), and the EEPS described below is going to act on stage 2 (*R ≥ ST*). According to the principle of SSA, the producers would move to a safer place when perceiving danger coming. In the new location update process, the producers would search globally with a normally distributed random manner and eventually converge to the optimal position. Nevertheless, there are still some limitations in this moving process, i.e., (i) the existing searching range could continue to be expanded; and (ii) the single global searching process could affect the convergence accuracy. Therefore, we design the EEPS to balance the exploration process and exploitation process in this paper. In fact, the balancing effect of EEPS is able to dynamically adjust the position searching approach of the producers, i.e., by promoting the producers exploring the whole space with a global searching approach for expanding the searching range of the potential optimal position in the early stage, while exploiting the current area with a local searching approach when a certain area is close to the optimal position.

Similarly, inspired by the literature [37,38] related to HGS, we introduce the pattern of food-approaching into the location update process of producers in stage 2, and construct the balance factor shown in Equation (14):

(14) $$balance\_factor_{iter}=\left(2para-1\right)\times \left(\delta \times \left(1-\frac{iter}{iter\_\max}\right)\right)$$

where the meaning of *para* is a random number in the range of (0, 1), the ***δ*** indicates a control parameter (it is set to 2), the *iter* indicates the current number of iterations, and the *iter_max* indicates the maximum number of iterations.

Subsequently, the EEPS, which combines the balance factor with the original location update mechanism, is shown in Equation (15):

(15) $$posi_{i,\;d}^{t+1}=\left(\left(2para-1\right)\times \left(\delta \times \left(1-\frac{iter}{iter\_\max}\right)\right)\right)\cdot^{*}\left(posi_{\mathrm{best}}^{t}-posi_{i,\;d}^{t}\right)+\mathrm{Random}\cdot Matrix\;R\;\geq \;ST$$

where the meaning of the parameters are similar to Equations (4), (6) and (14).

**Figure 3:**
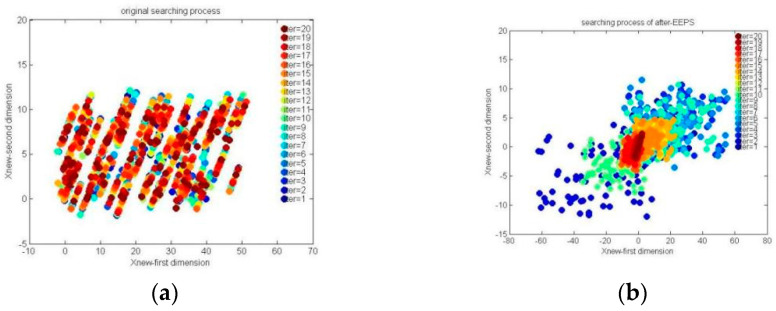
The comparison result figures of searching processes. (**a**) the original searching process; (**b**) the searching process based on after-EEPS.

The comparison results of the original searching process and the searching process based on after-EEPS are shown in Figure 3a,b, respectively.

In Figure 3, the points in the same color are the positions of all producers in one iteration, while different colors indicate different iterations. Figure 3a shows that the original location update mechanism of producers in stage 2 could achieve a global searching process to some extent, but it lacks the local exploitation behavior and the global searching range is not large enough, which can finally affect the convergence accuracy. As a comparison, Figure 3b shows that the EEPS not only expands the global searching range, but retains the exploitation ability, which can be clearly seen in the color changing process of points.

### 3.3. Perturbation–Exploration Strategy (PES)

Notably, the PHFS and EEPS only enhance the exploration ability of producer populations, which means that there remains the possibility of trapping into local optimum for the whole sparrow populations. Therefore, we introduce the Cauchy distribution operator [39,40,41] to construct the PES to further expand the searching range and avoid the local optimum situation in late iterations.

In the PES, we adopt the Cauchy distribution operator to obtain a variant of the current optimal individual. Moreover, we compare the fitness of the current optimal individual with that of the variant to save the better solution. In this paper, the probability density function of Cauchy distribution operator is shown as follows:

(16) $$cauchy\left(k\right)=\frac{1}{\pi }\cdot \frac{a}{a+k^{2}}$$

where the *a* is a parameter (in this paper, the *a* is equal to 1), and the *k* indicates a variable, the value range of which is from negative infinity to positive infinity. Based on the Cauchy distribution operator, we could obtain the perturbation–exploration process as follows:

(17) $$\left\{ \begin{array}{l} r=\tan\left(\left(\mathrm{random}-0.5\right)\times \pi \right)\\ {\mathrm{posi}}_{best}^{new}=posi_{best}^{t}+r\times posi_{best}^{t} \end{array}\right.$$

where the *r* is a Cauchy distribution random variable generating function.

### 3.4. Parameter Self-Adaptive Strategy (PSAS)

As we all know, obtaining the appropriate parameter values (*k* and *c*) is most significant for the original KELM. However, the existing method of parameter taking is to adopt grid searching, which not only increases the computational cost but also affects the final results of parameter obtaining. To achieve a self-adaptive process of these two parameters, we propose the PSAS via combining the EGSSA with the KELM.

Specifically, there are four significant stages in the PSAS, and the technical details are as follows: (i) achieving the location initialization process of sparrow populations (this paper adopts the random generation method of the original SSA); (ii) the core parameters (*k* and *c*) could be automatically obtained by adopting the EGSSA; (iii) to eliminate the randomness of these two obtained parameters, this paper utilizes 10-fold cross-validation [18] to re-obtain the optimal parameter values; and (iv) the re-obtained optimal parameter values are introduced into the original KELM, and finally the MsO-KELM is formed to perform the prediction performance on six different disease datasets based on 10-fold cross-validation.

In summary, the novel MsO-KELM model utilizes the four major strategies, i.e., PHFS, EEPS, PES, and PSAS, to achieve improved prediction performance, and the specific details of the MsO-KELM are shown in Algorithm 1.

**Algorithm 1:** MsO-KELM model

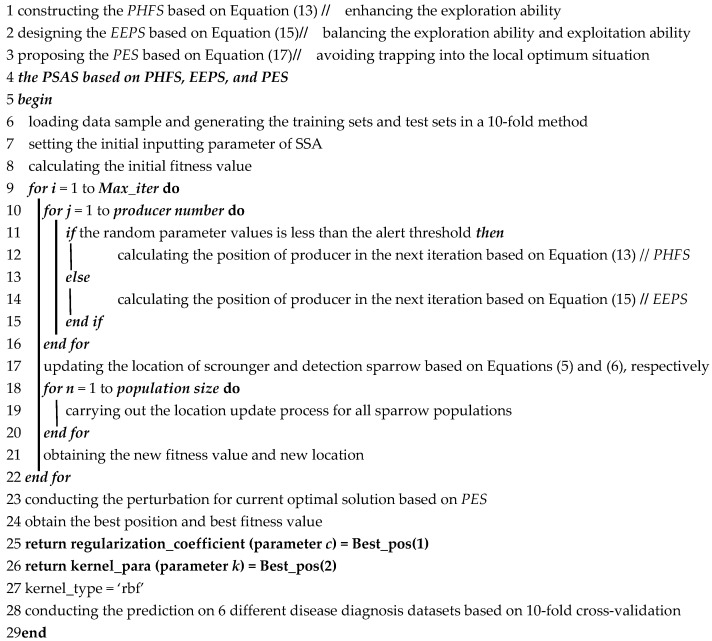



## 4. Results

In this section, we mainly analyze the results of two experiments, i.e., the performance analysis of the EGSSA and the disease prediction evaluation of the MsO-KELM. To ensure the reasonableness of these two experiments, we set the population size and the maximum iterations of each experiment group to (30, 500) and (10, 50), respectively. Importantly, each algorithm in experiment 1 runs 30 times independently on each function to minimize the effects of algorithmic randomness [30]. Moreover, all experiments are conducted in the same running environment, i.e., Intel Core i7, 2.40 GHz, 8 GB RAM, MATLAB2021a, and the IBM SPSS Statistics 20.

### 4.1. Experiment 1: The Performance Analysis of EGSSA

In this paper, we select nine different swarm intelligence algorithms to conduct the comparison analysis, which contain seven classical algorithms, i.e., SSA [29], PSO [42], GWO [43], HHO [44], LSA [45], WOA [46], and FPA [47], and two classical swarm intelligence variants, i.e., SCACSSA [48] and HHOHGSO [49]. The parameters which exist in both EGSSA and SSA are set to the same values, and the specific parameter settings in this experiment are shown in Table 2.

#### 4.1.1. Performance Analysis Based on 23 Classical Benchmark Functions

The 23 classical benchmark functions are frequently adopted to evaluate the performance of optimization algorithms, which could be divided into three categories, i.e., the unimodal functions, the multimodal functions, and the fixed-dimension multimodal functions [30]. The specific characteristics of these 23 benchmark functions are shown in Table 3, and the experimental results of the aforementioned algorithms based on the 23 functions are shown in Table 4.

In this experiment, considering that all the swarm intelligence optimization algorithms should perform multiple iterations, we utilize the mean value (*avg*) and the standard deviation value (*std*) to measure the performance [30], where the *avg* value is the key metric. The *avg* value being closer to the optimal value means the algorithm has a better performance in solving the current function. In addition, the *std* value could show the stability of this algorithm in solving the current function. Notably, when there are the same *avg* values between two algorithms, the *std* value could be the second evaluation metric.

According to the results in Table 4, we find that the EGSSA could obtain the best results in solving the benchmark functions F1, F2, F3, F4, F5, F6, F9, F10, F11, F12, F13, F16, F17, F18, F19, F20, F21, F22, and F23 among all 10 compared algorithms. Furthermore, in solving the unimodal functions F1-F4, the EGSSA not only obtains the optimal values, but also has the most stable performance. In solving the multimodal functions F9, F11, and the fixed-dimension multimodal functions F16-F19, the EGSSA can obtain the optimal values, while solving the multimodal functions F10, F12, F13, and the fixed-dimension multimodal functions F20–F23, the EGSSA can obtain the best results among all 10 compared algorithms and show more stable performance than others.

**Figure 4:**
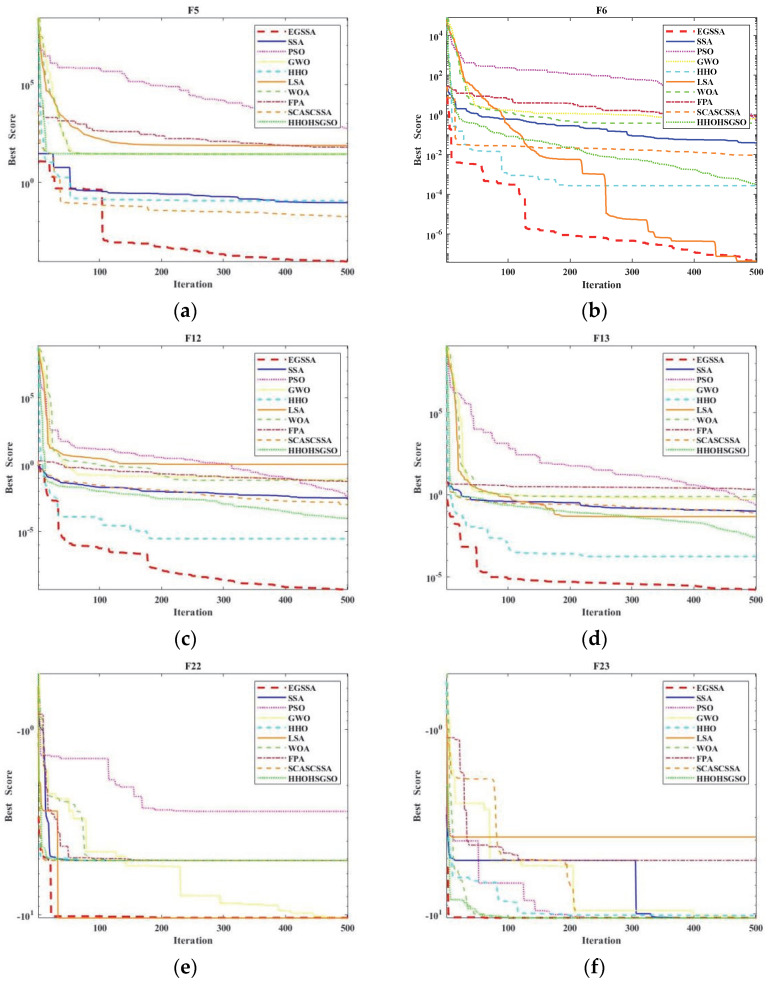
The convergence curves of EGSSA and other algorithms. (**a**) the convergence curves on F5; (**b**) the convergence curves on F6; (**c**) the convergence curves on F12; (**d**) the convergence curves on F13; (**e**) the convergence curves on F22; (**f**) the convergence curves on F23.

Apart from the comparison results table, we could verify the validity of EGSSA based on the convergence curve figure. In Figure 4, the F5 and F6 are unimodal functions, the F12 and F13 are multimodal functions, and the F22 and F23 are the fixed-dimension multimodal functions. According to Figure 4, it indicates that the EGSSA can obtain the fastest convergence among the 10 compared algorithms in solving the benchmark functions F6, F12, F13, F22, and F23. When solving the function F5, the convergence speed of EGSSA is similar to that of HHO, SSA, and SCASCSSA during the first 100 iterations, which is significantly superior to others, and the EGSSA can obtain the fastest convergence among the 10 compared algorithms after 100 iterations. In summary, the EGSSA enhances the overall convergence performance compared with the original SSA, other classical swarm intelligence optimization algorithms, and other variants. Therefore, the EGSSA could provide good support for the construction of the subsequent disease prediction model.

#### 4.1.2. Statistical Test

To show the statistical significance of the proposed EGSSA, we introduce the Wilcoxon rank-sum test into this section [30]. The better one of any two compared algorithms could be identified by comparing the obtained significance level value (*p*-value) with 0.05. The *p*-value being less than 0.05 indicates that the former (the algorithm to be verified) has more significant advantages than the latter (the algorithm being compared); otherwise, there is no significant difference between the latter and the former. The results of the statistical test are shown in Table 5.

According to Table 5, we find that the EGSSA could obtain 16 better statistical test results among 23 benchmark functions compared with the original SSA; meanwhile, it could also obtain 7 statistical test results which are equal to those of the original SSA. For the comparison results between the EGSSA and the other six classical swarm intelligence algorithms, it is clearly shown that the *p*-values of EGSSA versus PSO, EGSSA versus GWO, EGSSA versus HHO, EGSSA versus LSA, EGSSA versus WOA, and EGSSA versus FPA are much less than 0.05, and the EGSSA could obtain 16–19 better benchmark function results than the aforementioned compared algorithms. When comparing the EGSSA with the HHO, although the *p*-value is larger than 0.05, the number of benchmark functions which could obtain better results is still significantly higher than with the HHO. Similarly, for the comparison results between the EGSSA and the SCACSSA, we could find that the EGSSA has significant advantages compared with SCACSSA, and the former could obtain 19 better solving results among 23 benchmark functions while it could also obtain four statistical test results which are equal to those of the SCACSSA. When comparing the EGSSA with the HHOHGSO, although the *p*-value is larger than 0.05, the number of benchmark functions for which better results could be obtained is still significantly higher than the HHOHGSO.

### 4.2. Experiment 2: The Prediction Analysis in Solving Different Disease Datasets

**Figure 5:**
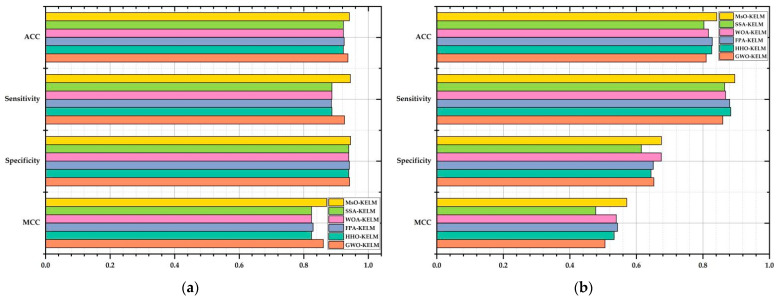
The figure of evaluation metric results on Breast Cancer and Parkinson. (**a**) the evaluation criterion result on Breast Cancer; (**b**) the evaluation criterion result on Parkinson.

**Figure 6:**
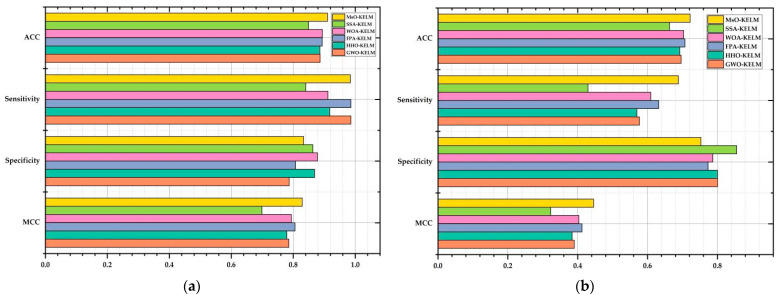
The figure of evaluation metric results on Autistic and Heart Disease. (**a**) the evaluation criterion result on Autistic; (**b**) the evaluation criterion result on Heart Disease.

**Figure 7:**
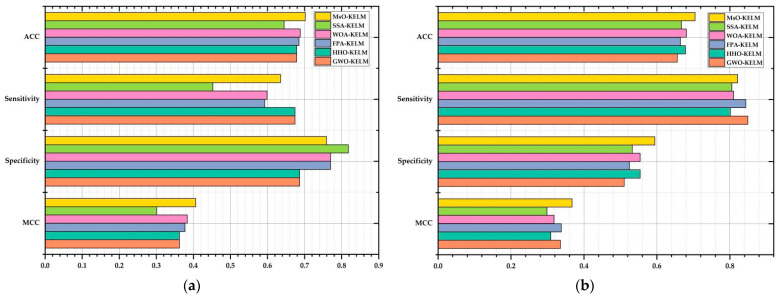
The figure of evaluation metric results on Cleveland and Bupa. (**a**) the evaluation criterion result on Cleveland; (**b**) the evaluation criterion result on Bupa.

This section is mainly to verify the prediction performance of the proposed MsO-KELM in solving the aforementioned six disease datasets. Furthermore, we select another five different optimization algorithms-based KELM variants to be the compared group, i.e., GWO-KELM, HHO-KELM, FPA-KELM, WOA-KELM, and SSA-KELM. The prediction results of different algorithms on four different evaluation metrics are shown in Table 6, Table 7, Table 8, Table 9, Table 10 and Table 11 and Figure 5, Figure 6 and Figure 7.

#### 4.2.1. Data Pre-Processing and Parameter Settings

Considering that the number of samples with missing values is about 1–3% of the sample volume of the relevant dataset, we therefore remove these samples with missing values. Meanwhile, we normalize all attributes to the interval form −1 to 1. To obtain a fair comparison result for these six different KELM variants, we set the same population size and maximum iteration numbers for all compared algorithms, to 10 and 50, respectively. In addition, to effectively estimate the generalization ability of the proposed model, we utilize 10-fold cross-validation to divide the original data samples into 10 groups, where each group can be used separately as a testing set, and the other 9 groups can be used as a training set, and, in that case, the 10 models can be obtained. These 10 models are evaluated in the 10 testing sets, respectively, and the final cross-validation result is obtained by summing and averaging the results of the 10 models. For the other special parameters, we can set the values based on Table 2.

#### 4.2.2. Experiment Results Analysis

The prediction results in solving Breast Cancer via 10-fold cross-validation method are shown in Table 6. Based on Table 6 and Figure 5a, it is clearly shown that the proposed MsO-KELM could obtain the best average values of ACC, sensitivity, specificity, and MCC among all six compared algorithms, at 94.124%, 94.434%, 94.539%, and 87.099%, respectively. As a comparison, the GWO-KELM obtains the second best average values of these four evaluation metrics at 93.695%, 92.628%, 94.212%, and 86.068%, respectively. In addition, the HHO-KELM, the WOA-KELM, and the SSA-KELM all obtain the worst ACC values, specificity values, and MCC values at 92.237%, 93.944%, and 82.433%, respectively, but the FPA-KELM obtains the worst sensitivity value at 88.539%. According to these evaluation metric results, we could find that the proposed MsO-KELM enhances the overall prediction performance and outperforms the other optimization algorithm-based KELM models in predicting breast cancer; therefore, the MsO-KELM could be adopted in the early detection of breast cancer.

The prediction results in solving Parkinson via 10-fold cross-validation method are shown in Table 7. Based on Table 7 and Figure 5b, it is clearly shown that the proposed MsO-KELM could obtain the best average values of ACC, sensitivity, specificity, and MCC among all six compared algorithms at 84.167%, 89.596%, 67.571%, and 57.140%, respectively. As a comparison, the FPA-KELM could obtain the second best ACC value at 82.833% and the second best MCC value at 54.365%; the HHO-KELM and the WOA-KELM could obtain the second best sensitivity value at 88.423% and the second best specificity value at 67.500%, respectively. In addition, the SSA-KELM obtained the worst ACC value, specificity value, and MCC value at 80.333%, 61.500%, and 47.749%, respectively, but the GWO-KELM obtained the worst sensitivity value at 85.991%. According to these evaluation metric results, we could find that the proposed MsO-KELM has better prediction performance than other compared models for Parkinson’s disease.

The prediction results in solving Autistic Spectrum Disorder Screening Data for Children via 10-fold cross-validation method are shown in Table 8. Based on Table 8 and Figure 6a, it is clearly shown that the proposed MsO-KELM could obtain the best average values of ACC and MCC among all six compared algorithms at 91.079% and 82.899%, respectively. As a comparison, the WOA-KELM obtained the second best ACC values at 89.433% and the FPA-KELM obtained the second best MCC values at 80.578%. In addition, for the sensitivity value and specificity value, the MsO-KELM could obtain the results at 98.424% and 83.379%, respectively, which are close to the best values for these two metrics. According to these evaluation metric results, we could find that the proposed MsO-KELM has the best prediction accuracy among these compared models in the diagnosis of autism spectrum disorders.

The prediction results in solving Heart Disease via 10-fold cross-validation method are shown in Table 9. Based on Table 9 and Figure 6b, it is clearly shown that the proposed MsO-KELM could obtain the best average values of ACC, sensitivity, and MCC among all six compared algorithms at 72.222%, 68.853%, and 44.557%, respectively. As a comparison, the FPA-KELM obtained the second best ACC value, sensitivity value, and MCC values at 70.741%, 63.198%, and 41.245%, respectively. In addition, the SSA-KELM obtained the worst ACC value, sensitivity value, and MCC value at 66.296%, 42.951%, and 32.280%, respectively. For the sensitivity value, the MsO-KELM could obtain a result which is close to that of the other models. According to these evaluation metric results, we could find that the proposed MsO-KELM has more advantages and could obtain a better classification performance than the other compared models.

The prediction results in solving Cleveland via 10-fold cross-validation method are shown in Table 10. Based on Table 10 and Figure 7a, it is clearly shown that the proposed MsO-KELM could obtain the best average values of ACC and MCC among all six compared algorithms at 70.184% and 40.608%, respectively. As a comparison, the WOA-KELM obtained the second best average values of ACC and MCC at 68.839% and 38.364%, respectively. In addition, the SSA-KELM could obtain the worst ACC value, specificity value, and MCC value at 64.517%, 45.252%, and 30.108%, respectively, but the GWO-KELM and the HHO-KELM obtained the worst sensitivity value at 68.668%. According to these evaluation metric results, we could find that the proposed MsO-KELM is superior to the others and could be adopted to diagnose the Cleveland heart disease.

The prediction results in solving Bupa via 10-fold cross-validation method are shown in Table 11. Based on Table 11 and Figure 7b, it is clearly shown that the proposed MsO-KELM could obtain the best average values of ACC, specificity, and MCC among all six compared algorithms at 70.476%, 59.423%, and 36.706%, respectively. As a comparison, the WOA-KELM could obtain the second best ACC value at 68.095%, the WOA-KELM and the HHO-KELM could obtain the second best specificity value at 55.407%, and the FPA-KELM could obtain the second best MCC value at 33.780%. For the sensitivity value, the MsO-KELM could obtain a result which is close to that of the other models. According to these evaluation metric results, we could find that the proposed MsO-KELM has more advantages in predicting liver disease than the other compared models.

## 5. Discussion

Combining information technology with medical information to realize the auxiliary diagnosis is a research trend in the digital health age; therefore, researchers have conducted a large number of prediction studies on common diseases by utilizing some machine learning models. However, the existing studies have weak generalization performance, which limits their further application for other diseases. In other words, these findings may obtain better prediction results in solving a certain disease but may obtain worse prediction results in solving other, different diseases. The reason why these findings have weak generalization performance is that the researchers mainly emphasize result-orientation for a certain disease, i.e., they focus on training with single disease data to obtain an effective prediction model which is suitable for the current disease. In fact, a prediction model with better generalization performance could help medical workers to diagnose various diseases. Therefore, exploring a prediction model with strong generalization ability has important theoretical and practical significance in the current digital health age.

### 5.1. Theoretical Significance

This paper is an attempt to explore a technology-oriented pathway for auxiliary diagnosis. On the one hand, our investigation adopts different disease data as the experimental objects, and aims to expand the application scope of the final findings by mining the internal characteristics of these different disease data, which could provide a novel research idea for researchers to eliminate the application limitations of the existing studies, and could provide effective theoretical guidance for researchers to realize comprehensive auxiliary diagnosis in facing various diseases.

On the other hand, utilizing an enhanced meta-heuristic algorithm to optimize the operating mechanism and inner structure of the original classifier is an efficient method to improve the performance of a model. The proposed MsO-KELM not only enhances the generalization ability and rapid learning ability of the original KELM classifier, but also realizes the self-adaptive process in predicting different diseases by introducing the EGSSA optimizer. The results of the evaluation metrics show that the MsO-KELM has significant advantages among all compared models in predicting the six diseases. Specifically, the MsO-KELM can obtain the best ACC value when predicting each disease, such that the ACC value is 94.124% in predicting breast cancer, the ACC value is 91.079% in predicting Autistic Spectrum Disorder, and the ACC value is 84.167% in predicting Parkinson’s disease. These ACC values could reflect the effectiveness of the MsO-KELM in disease prediction. Compared with some specific disease models [11,50], there may be a slight decrease in prediction accuracy of the MsO-KELM. However, according to the No Free Lunch (NFL) theory [51], although the MsO-KELM has slightly decreased accuracy in predicting a certain disease, it could predict more different diseases, which enhances the medical application value of the model.

### 5.2. Practical Significance

On the one hand, for the six different real-world diseases selected in this study, our investigation could assist doctors to diagnose or screen patients with related diseases, guide patients to prevent the diseases in a targeted manner, reduce the risk level of these diseases, and finally improve the survival quality of the patients.

On the other hand, there may be a consistent one-to-one match between the existing prediction models and their prediction targets, which means they will increase the economic cost and time cost for medical departments when analyzing different diseases. Therefore, a universal prediction model with strong generalization ability and robustness could predict more different diseases so as to improve the overall work efficiency of the medical workers.

### 5.3. Limitations

This study completes the construction of a self-adaptive prediction model in solving different diseases, but some limitations related to the MsO-KELM should be noted.

(i) In terms of model, although this paper presents four entirely novel optimization strategies to further optimize the prediction performance of the model, the performance improvement is accompanied by an increase in computational complexity, which is inevitable and could be comprehended according to the NFL theory. Therefore, we will further enhance the prediction performance of the MsO-KELM by redesigning a novel parameter optimization mechanism and optimizing the fundamental structure of the prediction model to reduce the computational complexity in the future studies.

(ii) In terms of data, on the one hand, the disease data analyzed in this paper are publicly available datasets, where each disease dataset has a limited sample size and some datasets even have specific geographical characteristics; on the other hand, the number of disease categories covered by the selected datasets are not enough. Therefore, we will increase the category of disease data and expand the sample size of disease data. In addition, we will also strive for collecting global disease data to eliminate the influence of the geographical factors.

## 6. Conclusions

To effectively predict more diseases and provide more comprehensive auxiliary diagnosis for medical workers, this paper proposes a novel disease prediction model, i.e., the MsO-KELM. There are two experiments conducted in this paper to elaborate the details of the MsO-KELM. The first experiment shows an optimization process for the EGSSA, which aims to construct a self-adaptive characteristic for the subsequent MsO-KELM model. The second experiment proves that the MsO-KELM has high accuracy, strong generalization ability, and strong robustness, which highlights the better disease prediction performance of the MsO-KELM. In the future, we will conduct in-depth research into optimizing the prediction model and expanding the disease data sample to achieve a further breakthrough in medical informatics.

## Figures and Tables

**Table 1:** The characteristics of the six disease datasets.

Datasets	Data Volume	Attributes	Missing Values	Positive Volume	Negative Volume
Breast cancer	699	9	16	458	241
Heart disease	270	13	0	150	120
Parkinson	195	23	0	147	48
Autistic Spectrum Disorder Screening Data	292	21	4	141	151
Cleveland	303	13	4	139	164
Bupa	345	6	0	145	200

**Table 2:** The parameter values of different experiments.

Algorithm	Parameter Values	Reference
SSA	*ST* = 0.8, *PD* = 20%, *SD* = 10%	[29]
PSO	*c_1_* = 2, *c*_2_ = 2, *V_max_* = 10	[42]
GWO	a = [2, 0]	[43]
HHO	No input parameters required	[44]
LSA	*ctime* = 10	[45]
WOA	*a* = [2 0], *a*_2_ = [−2 −1], *b* = 1	[46]
FPA	*p* = 0.5	[47]
SCACSSA	*ST* = 0.8, *PD* = 20%, *SD* = 10%, *a* = 2	[48]
HHOHGSO	*α* = 2, *β* = 2, *K* = 1, *M_1_* = 0.1, *M*_2_ = 0.2	[49]

**Table 3:** Characteristics of the 23 classical benchmark functions [30].

Function		Function Equationuation	Dim	Range	Optimal
Unimodal	F1	$f_{1}\left(x\right)=\overset{n}{\underset{i=1}{\sum }}x_{i}^{2}$	30	[−100, 100]	0
	F2	$f_{2}\left(x\right)=\overset{n}{\underset{i=1}{\sum }}\left|x_{i}\right|+\overset{n}{\underset{i=1}{\prod }}\left|x_{i}\right|$	30	[−10, 10]	0
	F3	$f_{3}\left(x\right)=\overset{n}{\underset{i=1}{\sum }}{\left(\overset{i}{\underset{j-1}{\sum }}x_{j}\right)}^{2}$	30	[−100, 100]	0
	F4	$f_{4}\left(x\right)=max_{i}\left\{\left|x_{i}\right|,1\leq i\leq n\right\}$	30	[−100, 100]	0
	F5	$f_{5}\left(x\right)=\overset{n-1}{\underset{i=1}{\sum }}\left[100{\left(x_{i+1}-x_{i}{}^{2}\right)}^{2}+{\left(x_{i}-1\right)}^{2}\right]$	30	[−30, 30]	0
	F6	$f_{6}\left(x\right)=\overset{n}{\underset{i=1}{\sum }}{\left(\left[x_{i}+0.5\right]\right)}^{2}$	30	[−100, 100]	0
	F7	$f_{7}\left(x\right)=\overset{n}{\underset{i=1}{\sum }}ix_{i}^{4}+random[0,1)$	30	[−1.28, 1.28]	0
Multimodal	F8	$f_{8}\left(x\right)=\overset{n}{\underset{i=1}{\sum }}-x_{i}sin\left(\sqrt{\left|x_{i}\right|}\right)$	30	[−500, 500]	−418.9829 × 5
	F9	$f_{9}\left(x\right)=\overset{n}{\underset{i=1}{\sum }}\left[x_{i}^{2}-10cos\left(2\pi x_{i}\right)+10\right]$	30	[−5.12, 5.12]	0
	F10	$f_{10}\left(x\right)=-20exp\left(-0.2\sqrt{\frac{1}{n}\overset{n}{\underset{i=1}{\sum }}x^{i}}\right)-exp\left(\frac{1}{n}\overset{n}{\underset{i=1}{\sum }}cos\left(2\pi x_{i}\right)\right)+20+e$	30	[−32, 32]	0
	F11	$f_{11}\left(x\right)=\frac{1}{4000}\overset{n}{\underset{i=1}{\sum }}x_{i}^{2}-\overset{n}{\underset{i=1}{\prod }}cos\left(\frac{x_{i}}{\sqrt{i}}\right)+1$	30	[−600, 600]	0
	F12	$f_{12}\left(x\right)=\frac{\pi }{n}\left\{10sin\left(\pi y_{1}\right)+\overset{n-1}{\underset{i=1}{\sum }}{\left(y_{i}-1\right)}^{2}\left[1+10sin^{2}\left(\pi y_{i+1}\right)\right]+{\left(y_{n}-1\right)}^{2}\}+\overset{n}{\underset{i=1}{\sum }}\mu \left(x_{i},10,100,4\right)\right\}$	30	[−50, 50]	0
		$y_{i}=1+\frac{x_{i}+1}{4}$			
		$\mu \left(x_{i},a,k,m\right)=\{\begin{array}{c} k{\left(x_{i}-a\right)}^{m\;\;\;\;\;\;\;\;\;\;\;\;\;\;\;\;\;\;}\;\;\;x_{i}>a\\ 0\;\;\;\;\;\;\;\;\;\;\;\;\;\;\;\;\;\;\;-a<x_{i}<a\\ k{\left(-x_{i}-a\right)}^{m}\;\;\;\;\;\;\;\;x_{i}<-a \end{array}$			
	F13	$f_{13}\left(x\right)=0.1\left\{sin^{2}\left(3\pi x_{1}\right)+\overset{n}{\underset{i=1}{\sum }}{\left(x_{i}-1\right)}^{2}\left[1+sin^{2}\left(3\pi x_{i}+1\right)\right]+{\left(x_{n}-1\right)}^{2}\left[1+sin^{2}\left(2\pi x_{n}\right)\right]\right\}+\overset{n}{\underset{i=1}{\sum }}\mu \left(x_{i},5,100,4\right)$	30	[−50, 50]	0
Fixed-dimension multimodal	F14	$f_{14}\left(x\right)={\left(\frac{1}{500}+\overset{25}{\underset{j=1}{\sum }}\frac{1}{j+\sum_{i=1}^{2}{\left(x_{i}-a_{ij}\right)}^{6}}\right)}^{-1}$	2	[−65, 65]	1
	F15	$f_{15}\left(x\right)=\overset{11}{\underset{i=1}{\sum }}{\left[a_{i}-\frac{x_{1}\left(b_{i}{}^{2}+b_{i}x_{2}\right)}{b_{i}{}^{2}+b_{i}x_{3}+x_{4}}\right]}^{2}$	4	[−5, 5]	0.00030
	F16	$f_{16}\left(x\right)=4x_{1}{}^{2}-2.1x_{i}{}^{4}+\frac{1}{3}x_{1}{}^{6}+x_{1}x_{2}-4x_{2}{}^{2}+4x_{2}{}^{4}$	2	[−5, 5]	−1.0316
	F17	$f_{17}\left(x\right)={\left(x_{2}-\frac{5.1}{4\pi^{2}}x_{1}{}^{2}+\frac{5}{\pi }x_{1}-6\right)}^{2}+10\left(1-\frac{1}{8\pi }\right)cosx_{1}+10$	2	[−5, 5]	0.398
	F18	$f_{18}\left(x\right)=\left[1+{\left(x_{1}+x_{2}+1\right)}^{2}\left(19-14x_{1}+3x_{1}{}^{2}-14x_{2}+6x_{1}x_{2}+3x_{2}{}^{2}\right)\right]\times \left[30+{\left(2x_{1}-3x_{2}\right)}^{2}\times \left(18-32x_{1}+12x_{1}{}^{2}+48x_{2}-36x_{1}x_{2}+27x_{2}{}^{2}\right)\right]$	2	[−2, 2]	3
	F19	$f_{19}\left(x\right)=-\overset{4}{\underset{i=1}{\sum }}c_{i}exp\left(-\overset{3}{\underset{j=1}{\sum }}a_{ij}{\left(x_{i}-p_{ij}\right)}^{2}\right)$	3	[1, 3]	−3.86
	F20	$f_{20}\left(x\right)=-\overset{4}{\underset{i=1}{\sum }}c_{i}exp\left(-\overset{6}{\underset{j=1}{\sum }}a_{ij}{\left(x_{i}-p_{ij}\right)}^{2}\right)$	6	[0, 1]	−3.32
	F21	$f_{21}\left(x\right)=-\overset{5}{\underset{i=1}{\sum }}{\left[\left(X-a_{i}\right){\left(X-a_{i}\right)}^{T}+c_{i}\right]}^{-1}$	4	[0, 10]	−10.1532
	F22	$f_{22}\left(x\right)=-\overset{7}{\underset{i=1}{\sum }}{\left[\left(X-a_{i}\right){\left(X-a_{i}\right)}^{T}+c_{i}\right]}^{-1}$	4	[0, 10]	−10.4028
	F23	$f_{23}\left(x\right)=-\overset{10}{\underset{i=1}{\sum }}{\left[\left(X-a_{i}\right){\left(X-a_{i}\right)}^{T}+c_{i}\right]}^{-1}$	4	[0, 10]	−10.5363

**Table 4:** The results based on the 23 classical benchmark functions (retaining two decimal places).

		EGSSA	SSA	PSO	GWO	HHO	LSA	WOA	FPA	SCACSSA	HHOHGSO
F1	avg	0.00 × 10^00^	1.30 × 10^−49^	6.25 × 10^−01^	1.19 × 10^−27^	1.61 × 10^−93^	1.11 × 10^−03^	7.15 × 10^−72^	4.60 × 10^−01^	6.17 × 10^−16^	5.82 × 10^−268^
	std	0.00 × 10^00^	7.11 × 10^−49^	3.36 × 10^−01^	1.64 × 10^−27^	8.22 × 10^−93^	5.93 × 10^−03^	3.90 × 10^−71^	1.39 × 10^−01^	3.35 × 10^−15^	0.00 × 10^00^
F2	avg	0.00 × 10^00^	5.36 × 10^−27^	3.26 × 10^01^	1.28 × 10^−16^	2.34 × 10^−48^	2.48 × 10^−01^	9.69 × 10^−51^	2.80 × 10^00^	1.53 × 10^−07^	9.93 × 10^−160^
	std	0.00 × 10^00^	2.90 × 10^−26^	5.50 × 10^01^	1.32 × 10^−16^	1.25 × 10^−47^	3.80 × 10^−01^	4.84 × 10^−50^	3.64 × 10^−01^	6.56 × 10^−07^	5.44 × 10^−159^
F3	avg	0.00 × 10^00^	2.61 × 10^−29^	3.37 × 10^02^	1.43 × 10^−05^	7.32 × 10^−64^	1.37 × 10^02^	4.99 × 10^04^	3.28 × 10^−01^	1.76 × 10^−08^	2.17 × 10^−309^
	std	0.00 × 10^00^	1.18 × 10^−28^	9.30 × 10^01^	3.34 × 10^−05^	4.01 × 10^−63^	8.69 × 10^01^	1.66 × 10^04^	1.02 × 10^−01^	5.08 × 10^−08^	0.00 × 10^00^
F4	avg	0.00 × 10^00^	3.66 × 10^−26^	2.77 × 10^00^	7.27 × 10^−07^	9.66 × 10^−49^	9.62 × 10^00^	5.04 × 10^01^	3.67 × 10^−01^	9.26 × 10^−26^	5.81 × 10^−147^
	std	0.00 × 10^00^	1.99 × 10^−25^	4.30 × 10^−01^	1.06 × 10^−06^	4.89 × 10^−48^	4.25 × 10^00^	2.86 × 10^01^	4.42 × 10^−02^	4.86 × 10^−25^	3.18 × 10^−146^
F5	avg	2.56 × 10^−02^	4.61 × 10^−01^	6.55 × 10^02^	2.73 × 10^01^	1.01 × 10^−02^	1.21 × 10^02^	2.80 × 10^01^	7.96 × 10^01^	1.03 × 10^00^	2.73 × 10^01^
	std	9.08 × 10^−02^	7.42 × 10^−01^	4.83 × 10^02^	8.13 × 10^−01^	9.64 × 10^−03^	1.84 × 10^02^	4.42 × 10^−01^	1.81 × 10^01^	1.56 × 10^00^	6.72 × 10^−01^
F6	avg	9.24 × 10^−06^	3.05 × 10^−02^	6.67 × 10^−01^	6.92 × 10^−01^	2.90 × 10^−04^	7.36 × 10^−05^	3.94 × 10^−01^	1.23 × 10^00^	2.35 × 10^−02^	1.13 × 10^−03^
	std	1.38 × 10^−05^	1.64 × 10^−02^	3.37 × 10^−01^	3.65 × 10^−01^	6.00 × 10^−04^	3.71 × 10^−04^	2.32 × 10^−01^	3.99 × 10^−01^	1.14 × 10^−02^	1.27 × 10^−03^
F7	avg	1.11 × 10^−04^	7.70 × 10^−04^	2.52 × 10^00^	2.25 × 10^−03^	1.90 × 10^−04^	3.05 × 10^−02^	3.59 × 10^−03^	5.52 × 10^−01^	6.41 × 10^−02^	9.76 × 10^−05^
	std	1.46 × 10^−04^	6.79 × 10^−04^	2.80 × 10^00^	1.12 × 10^−03^	2.11 × 10^−04^	8.45 × 10^−03^	5.59 × 10^−03^	2.76 × 10^−01^	5.67 × 10^−02^	9.71 × 10^−05^
F8	avg	−8.10 × 10^03^	−7.79 × 10^03^	−6.63 × 10^03^	−5.89 × 10^03^	−1.26 × 10^04^	−7.57 × 10^03^	−1.02 × 10^04^	−4.26 × 10^01^	−6.04 × 10^03^	−1.13 × 10^04^
	std	2.23 × 10^03^	3.03 × 10^03^	7.63 × 10^02^	1.07 × 10^03^	1.40 × 10^00^	7.48 × 10^02^	1.70 × 10^03^	2.65 × 10^00^	7.52 × 10^02^	1.19 × 10^03^
F9	avg	0.00 × 10^00^	0.00 × 10^00^	1.43 × 10^02^	1.92 × 10^00^	0.00 × 10^00^	6.92 × 10^01^	0.00 × 10^00^	4.22 × 10^01^	3.46 × 10^−10^	0.00 × 10^00^
	std	0.00 × 10^00^	0.00 × 10^00^	3.74 × 10^01^	3.27 × 10^00^	0.00 × 10^00^	1.60 × 10^01^	0.00 × 10^00^	1.93 × 10^01^	1.53 × 10^−09^	0.00 × 10^00^
F10	avg	8.88 × 10^−16^	8.88 × 10^−16^	1.85 × 10^00^	1.04 × 10^−13^	8.88 × 10^−16^	2.90 × 10^00^	3.85 × 10^−15^	1.38 × 10^00^	2.35 × 10^−07^	8.88 × 10^−16^
	std	0.00 × 10^00^	0.00 × 10^00^	7.06 × 10^−01^	1.83 × 10^−14^	0.00 × 10^00^	8.34 × 10^−01^	2.30 × 10^−15^	2.31 × 10^−01^	1.28 × 10^−06^	0.00 × 10^00^
F11	avg	0.00 × 10^00^	0.00 × 10^00^	6.22 × 10^−02^	4.49 × 10^−03^	0.00 × 10^00^	7.22 × 10^−03^	5.86 × 10^−03^	1.66 × 10^−02^	3.20 × 10^−11^	0.00 × 10^00^
	std	0.00 × 10^00^	0.00 × 10^00^	4.06 × 10^−02^	8.49 × 10^−03^	0.00 × 10^00^	1.09 × 10^−02^	3.21 × 10^−02^	5.72 × 10^−03^	1.76 × 10^−10^	0.00 × 10^00^
F12	avg	1.43 × 10^−07^	1.00 × 10^−02^	1.17 × 10^00^	3.97 × 10^−02^	1.01 × 10^−05^	6.76 × 10^−01^	2.70 × 10^−02^	6.34 × 10^−02^	2.15 × 10^−03^	1.00 × 10^−04^
	std	1.18 × 10^−07^	3.16 × 10^−02^	2.30 × 10^00^	1.49 × 10^−02^	1.27 × 10^−05^	1.41 × 10^00^	1.61 × 10^−02^	2.52 × 10^−02^	1.27 × 10^−03^	8.86 × 10^−05^
F13	avg	6.36 × 10^−05^	2.53 × 10^−01^	4.69 × 10^−01^	7.10 × 10^−01^	8.67 × 10^−05^	6.36 × 10^−02^	5.62 × 10^−01^	1.07 × 10^00^	1.41 × 10^−01^	1.88 × 10^−02^
	std	1.74 × 10^−04^	1.31 × 10^−01^	3.07 × 10^−01^	2.93 × 10^−01^	1.02 × 10^−04^	1.32 × 10^−01^	3.28 × 10^−01^	3.07 × 10^−01^	1.06 × 10^−01^	1.72 × 10^−02^
F14	avg	9.95 × 10^00^	1.09 × 10^01^	3.33 × 10^00^	4.52 × 10^00^	1.26 × 10^00^	1.36 × 10^00^	3.00 × 10^00^	1.27 × 10^01^	1.27 × 10^01^	1.03 × 10^00^
	std	4.04 × 10^00^	3.83 × 10^00^	2.81 × 10^00^	4.22 × 10^00^	9.32 × 10^−01^	1.02 × 10^00^	3.06 × 10^00^	1.34 × 10^−14^	9.61 × 10^−11^	1.81 × 10^−01^
F15	avg	3.58 × 10^−04^	4.79 × 10^−04^	8.90 × 10^−04^	3.05 × 10^−03^	3.81 × 10^−04^	5.94 × 10^−04^	1.17 × 10^−03^	3.08 × 10^−04^	4.85 × 10^−04^	4.08 × 10^−04^
	std	8.86 × 10^−05^	1.46 × 10^−04^	1.33 × 10^−04^	6.91 × 10^−03^	2.13 × 10^−04^	3.25 × 10^−04^	2.43 × 10^−03^	1.19 × 10^−06^	2.36 × 10^−04^	2.40 × 10^−04^
F16	avg	−1.03 × 10^00^	−1.03 × 10^00^	−1.03 × 10^00^	−1.03 × 10^00^	−1.03 × 10^00^	−1.03 × 10^00^	−1.03 × 10^00^	−1.03 × 10^00^	−1.03 × 10^00^	−1.03 × 10^00^
	std	3.00 × 10^−10^	4.74 × 10^−16^	5.30 × 10^−16^	1.45 × 10^−08^	3.95 × 10^−10^	6.58 × 10^−16^	9.97 × 10^−10^	1.53 × 10^−09^	2.47 × 10^−03^	7.54 × 10^−13^
F17	avg	3.98 × 10^−01^	3.98 × 10^−01^	3.98 × 10^−01^	3.98 × 10^−01^	3.98 × 10^−01^	3.98 × 10^−01^	3.98 × 10^−01^	7.78 × 10^00^	3.98 × 10^−01^	3.98 × 10^−01^
	std	2.09 × 10^−04^	8.68 × 10^−09^	0.00 × 10^00^	6.52 × 10^−07^	2.87 × 10^−06^	0.00 × 10^00^	1.26 × 10^−05^	2.71 × 10^−15^	1.23 × 10^−06^	2.63 × 10^−10^
F18	avg	3.00 × 10^00^	3.00 × 10^00^	3.00 × 10^00^	3.00 × 10^00^	3.00 × 10^00^	3.00 × 10^00^	3.00 × 10^00^	3.00 × 10^00^	3.00 × 10^00^	3.00 × 10^00^
	std	2.85 × 10^−05^	9.65 × 10^−15^	4.41 × 10^−15^	4.39 × 10^−05^	3.54 × 10^−07^	2.56 × 10^−15^	9.64 × 10^−05^	2.14 × 10^−10^	6.60 × 10^−04^	1.03 × 10^−11^
F19	avg	−3.86 × 10^00^	−3.86 × 10^00^	−3.86 × 10^00^	−3.86 × 10^00^	−3.86 × 10^00^	−3.86 × 10^00^	−3.86 × 10^00^	−3.86 × 10^00^	−3.86 × 10^00^	−3.86 × 10^00^
	std	1.36 × 10^−04^	2.46 × 10^−05^	2.36 × 10^−15^	1.91 × 10^−03^	5.19 × 10^−03^	2.67 × 10^−15^	7.91 × 10^−03^	6.33 × 10^−09^	2.77 × 10^−05^	1.09 × 10^−05^
F20	avg	−3.29 × 10^00^	−3.26 × 10^00^	−3.27 × 10^00^	−3.27 × 10^00^	−3.07 × 10^00^	−3.27 × 10^00^	−3.20 × 10^00^	−3.28 × 10^00^	−3.25 × 10^00^	−3.25 × 10^00^
	std	5.33 × 10^−02^	6.84 × 10^−02^	6.03 × 10^−02^	7.31 × 10^−02^	1.26 × 10^−01^	5.92 × 10^−02^	1.56 × 10^−01^	3.26 × 10^−02^	6.29 × 10^−02^	8.31 × 10^−02^
F21	avg	−9.85 × 10^00^	−8.24 × 10^00^	−7.14 × 10^00^	−9.31 × 10^00^	−5.21 × 10^00^	−7.72 × 10^00^	−8.65 × 10^00^	−5.06 × 10^00^	−8.79 × 10^00^	−6.58 × 10^00^
	std	5.45 × 10^−01^	2.46 × 10^00^	3.38 × 10^00^	1.92 × 10^00^	8.73 × 10^−01^	3.13 × 10^00^	2.85 × 10^00^	2.94 × 10^−07^	2.29 × 10^00^	2.38 × 10^00^
F22	avg	−1.02 × 10^01^	−9.16 × 10^00^	−6.19 × 10^00^	−1.02 × 10^01^	−5.23 × 10^00^	−8.11 × 10^00^	−6.97 × 10^00^	−5.09 × 10^00^	−8.81 × 10^00^	−6.68 × 10^00^
	std	4.27 × 10^−01^	2.29 × 10^00^	3.39 × 10^00^	9.70 × 10^−01^	8.01 × 10^−01^	3.34 × 10^00^	3.36 × 10^00^	2.36 × 10^−07^	2.48 × 10^00^	2.48 × 10^00^
F23	avg	−1.02 × 10^01^	−8.73 × 10^00^	−8.59 × 10^00^	−1.01 × 10^01^	−5.48 × 10^00^	−7.27 × 10^00^	−6.77 × 10^00^	−5.13 × 10^00^	−9.64 × 10^00^	−6.39 × 10^00^
	std	7.11 × 10^−01^	2.59 × 10^00^	3.34 × 10^00^	1.75 × 10^00^	1.34 × 10^00^	3.85 × 10^00^	3.23 × 10^00^	3.42 × 10^−07^	2.05 × 10^00^	2.33 × 10^00^

**Table 5:** The results of the statistical test.

Algorithm	Better	Equationual	Worst	W+	W−	*p*-Value
EGSSA versus SSA	16	7	0	136	0	0.000438
EGSSA versus PSO	18	4	1	176	14	0.001116
EGSSA versus GWO	17	5	1	155	16	0.002472
EGSSA versus HHO	13	7	3	95	41	0.162673
EGSSA versus LSA	18	4	1	176	14	0.001116
EGSSA versus WOA	16	5	2	140	31	0.017621
EGSSA versus FPA	19	3	1	209	1	0.000103
EGSSA versus SCACSSA	19	4	0	190	0	0.000132
EGSSA versus HHOHGSO	12	8	3	88	32	0.111769

**Table 6:** The prediction results on disease dataset Breast Cancer.

Breast Cancer													
Indicator	Algorithms	Mean	Std	1#	2#	3#	4#	5#	6#	7#	8#	9#	10#
ACC	GWO-KELM	0.93695	0.03847	0.86765	0.95588	0.95588	0.97059	0.92647	0.86765	0.92647	0.98529	0.95588	0.95775
	HHO-KELM	0.92237	0.04657	0.83824	0.92647	0.94118	0.97059	0.95588	0.85294	0.88235	0.98529	0.94118	0.92958
	FPA-KELM	0.92531	0.04580	0.85294	0.92647	0.94118	0.98529	0.95588	0.85294	0.88235	0.98529	0.94118	0.92958
	WOA-KELM	0.92237	0.04657	0.83824	0.92647	0.94118	0.97059	0.95588	0.85294	0.88235	0.98529	0.94118	0.92958
	SSA-KELM	0.92237	0.04657	0.83824	0.92647	0.94118	0.97059	0.95588	0.85294	0.88235	0.98529	0.94118	0.92958
	MsO-KELM	0.94124	0.03785	0.85294	0.95588	0.95588	0.97059	0.92647	0.89706	0.94118	0.95588	0.97059	0.98592
Sensitivity	GWO-KELM	0.92628	0.06586	0.79412	0.92593	0.92308	1.00000	0.96970	1.00000	0.89474	1.00000	0.90909	0.84615
	HHO-KELM	0.88751	0.11278	0.73529	0.92593	0.92308	0.97368	1.00000	1.00000	0.68421	1.00000	0.86364	0.76923
	FPA-KELM	0.88539	0.12182	0.76471	0.92593	0.92308	1.00000	1.00000	1.00000	0.68421	1.00000	0.86364	0.69231
	WOA-KELM	0.88751	0.11278	0.73529	0.92593	0.92308	0.97368	1.00000	1.00000	0.68421	1.00000	0.86364	0.76923
	SSA-KELM	0.88751	0.11278	0.73529	0.92593	0.92308	0.97368	1.00000	1.00000	0.68421	1.00000	0.86364	0.76923
	MsO-KELM	0.94434	0.06814	0.76471	0.96296	0.92308	0.97368	0.9697	1.00000	0.89474	1.00000	0.95455	1.00000
Specificity	GWO-KELM	0.94212	0.04838	0.94118	0.97561	0.97619	0.93333	0.88571	0.82692	0.93878	0.98246	0.97826	0.98276
	HHO-KELM	0.93944	0.04847	0.94118	0.92683	0.95238	0.96667	0.91429	0.80769	0.95918	0.98246	0.97826	0.96552
	FPA-KELM	0.94117	0.04965	0.94118	0.92683	0.95238	0.96667	0.91429	0.80769	0.95918	0.98246	0.97826	0.98276
	WOA-KELM	0.93944	0.04847	0.94118	0.92683	0.95238	0.96667	0.91429	0.80769	0.95918	0.98246	0.97826	0.96552
	SSA-KELM	0.93944	0.04847	0.94118	0.92683	0.95238	0.96667	0.91429	0.80769	0.95918	0.98246	0.97826	0.96552
	MsO-KELM	0.94539	0.03753	0.94118	0.95122	0.97619	0.96667	0.88571	0.86538	0.95918	0.94737	0.97826	0.98276
MCC	GWO-KELM	0.86068	0.07295	0.74338	0.90771	0.90635	0.94163	0.85652	0.72748	0.82083	0.94899	0.89852	0.85542
	HHO-KELM	0.82433	0.09736	0.69128	0.84779	0.87546	0.94035	0.91548	0.70501	0.69626	0.94899	0.86440	0.75824
	FPA-KELM	0.82916	0.09867	0.71714	0.84779	0.87546	0.97051	0.91548	0.70501	0.69626	0.94899	0.86440	0.75053
	WOA-KELM	0.82433	0.09736	0.69128	0.84779	0.87546	0.94035	0.91548	0.70501	0.69626	0.94899	0.86440	0.75824
	SSA-KELM	0.82433	0.09736	0.69128	0.84779	0.87546	0.94035	0.91548	0.70501	0.69626	0.94899	0.86440	0.75824
	MsO-KELM	0.87099	0.071906	0.71714	0.90886	0.90635	0.94035	0.85652	0.77589	0.85392	0.86276	0.93281	0.95528

**Table 7:** The prediction results on disease dataset Parkinson.

Parkinson													
Indicator	Algorithms	Mean	Std	1#	2#	3#	4#	5#	6#	7#	8#	9#	10#
ACC	GWO-KELM	0.81000	0.06633	0.75000	0.75000	0.80000	0.75000	0.90000	0.85000	0.80000	0.95000	0.75000	0.80000
	HHO-KELM	0.82667	0.08206	0.80000	0.65000	0.80000	0.95000	0.85000	0.80000	0.90000	0.75000	0.90000	0.86667
	FPA-KELM	0.82833	0.06103	0.75000	0.80000	0.90000	0.75000	0.80000	0.80000	0.80000	0.85000	0.90000	0.93333
	WOA-KELM	0.81667	0.05164	0.85000	0.85000	0.80000	0.80000	0.85000	0.75000	0.85000	0.85000	0.70000	0.86667
	SSA-KELM	0.80333	0.08021	0.90000	0.85000	0.90000	0.65000	0.85000	0.70000	0.80000	0.85000	0.80000	0.73333
	MsO-KELM	0.84167	0.07042	0.85000	0.95000	0.85000	0.90000	0.75000	0.85000	0.70000	0.80000	0.90000	0.86667
Sensitivity	GWO-KELM	0.85991	0.09367	0.75000	0.75000	0.84615	0.76471	1.00000	0.92308	0.78571	1.00000	0.93333	0.84615
	HHO-KELM	0.88423	0.10233	0.88235	0.80000	0.76471	1.00000	0.93333	0.92857	0.93333	0.66667	0.93333	1.00000
	FPA-KELM	0.88071	0.09250	0.76923	0.81250	0.92857	0.70588	0.87500	0.92857	0.84615	0.94118	1.00000	1.00000
	WOA-KELM	0.86809	0.10697	0.93750	0.82353	1.00000	0.69231	0.92857	0.81250	1.00000	0.88235	0.68750	0.91667
	SSA-KELM	0.86521	0.10720	0.93750	1.00000	0.93750	0.66667	0.93750	0.73333	0.93750	0.92857	0.82353	0.75000
	MsO-KELM	0.89596	0.068495	0.87500	0.93333	0.84615	0.88889	0.80000	0.87500	0.80000	1.00000	0.94118	1.00000
Specificity	GWO-KELM	0.65286	0.17666	0.75000	0.75000	0.71429	0.66667	0.60000	0.71429	0.83333	0.80000	0.20000	0.50000
	HHO-KELM	0.64417	0.27719	0.33333	0.20000	1.00000	0.87500	0.60000	0.50000	0.80000	1.00000	0.80000	0.33333
	FPA-KELM	0.65119	0.18505	0.71429	0.75000	0.83333	1.00000	0.50000	0.50000	0.71429	0.33333	0.66667	0.50000
	WOA-KELM	0.67500	0.18703	0.50000	1.00000	0.42857	1.00000	0.66667	0.50000	0.57143	0.66667	0.75000	0.66667
	SSA-KELM	0.61500	0.14092	0.75000	0.70000	0.75000	0.60000	0.50000	0.60000	0.25000	0.66667	0.66667	0.66667
	MsO-KELM	0.67571	0.24685	0.75000	1.00000	0.85714	1.00000	0.60000	0.75000	0.60000	0.20000	0.66667	0.33333
MCC	GWO-KELM	0.50579	0.19972	0.41931	0.41931	0.56044	0.33612	0.72761	0.66339	0.57907	0.86603	0.19245	0.29417
	HHO-KELM	0.53338	0.24618	0.21569	0.00000	0.57248	0.89872	0.57735	0.49099	0.73333	0.57735	0.73333	0.53452
	FPA-KELM	0.54365	0.14232	0.47076	0.49099	0.76190	0.51450	0.37500	0.49099	0.56044	0.32673	0.76376	0.68139
	WOA-KELM	0.53987	0.12579	0.49010	0.64169	0.57248	0.66375	0.62994	0.28868	0.68139	0.49010	0.35722	0.58333
	SSA-KELM	0.47749	0.18407	0.68750	0.73380	0.68750	0.23570	0.49010	0.30261	0.25000	0.62994	0.40423	0.35355
	MsO-KELM	0.57140	0.14677	0.57735	0.88192	0.68474	0.66667	0.37796	0.57735	0.40825	0.39736	0.60784	0.53452

**Table 8:** The prediction results on disease dataset Autistic Spectrum Disorder Screening Data for Children.

Autistic Spectrum Disorder Screening Data for Children													
Indicator	Algorithms	Mean	Std	1#	2#	3#	4#	5#	6#	7#	8#	9#	10#
ACC	GWO-KELM	0.88665	0.04423	0.89655	0.93103	0.89655	0.82759	0.86207	0.89655	0.93103	0.79310	0.89655	0.93548
	HHO-KELM	0.88643	0.04925	0.89655	0.89655	0.82759	0.93103	0.86207	0.86207	0.79310	0.89655	0.93103	0.96774
	FPA-KELM	0.89377	0.06262	0.86207	0.93103	0.82759	0.79310	0.93103	1.00000	0.96552	0.89655	0.82759	0.90323
	WOA-KELM	0.89422	0.05088	0.96552	0.89655	0.86207	0.89655	0.93103	0.89655	0.89655	0.96552	0.79310	0.83871
	SSA-KELM	0.84917	0.05838	0.86207	0.93103	0.79310	0.96552	0.82759	0.79310	0.79310	0.86207	0.79310	0.87097
	MsO-KELM	0.91079	0.05621	0.89655	0.96552	0.89655	0.93103	0.96552	0.93103	0.93103	0.75862	0.89655	0.93548
Sensitivity	GWO-KELM	0.98606	0.02807	1.00000	0.92308	1.00000	1.00000	1.00000	1.00000	1.00000	0.93750	1.00000	1.00000
	HHO-KELM	0.91786	0.08472	0.93750	0.92857	0.80000	1.00000	0.78947	0.92308	0.80000	1.00000	1.00000	1.00000
	FPA-KELM	0.98619	0.02764	1.00000	1.00000	1.00000	1.00000	0.92857	1.00000	1.00000	1.00000	0.93333	1.00000
	WOA-KELM	0.91246	0.08829	0.93750	0.92857	0.90909	0.93333	1.00000	0.86667	0.85714	1.00000	0.69231	1.00000
	SSA-KELM	0.84079	0.09657	0.83333	1.00000	0.72222	1.00000	0.87500	0.71429	0.75000	0.81250	0.81818	0.88235
	MsO-KELM	0.98424	0.03198	0.90909	1.00000	1.00000	1.00000	1.00000	1.00000	1.00000	1.00000	1.00000	0.93333
Specificity	GWO-KELM	0.78695	0.09798	0.82353	0.93750	0.78571	0.64286	0.71429	0.76923	0.86667	0.61538	0.85714	0.85714
	HHO-KELM	0.86932	0.05986	0.84615	0.86667	0.85714	0.87500	1.00000	0.81250	0.78571	0.83333	0.86667	0.95000
	FPA-KELM	0.80771	0.10983	0.77778	0.84615	0.70588	0.64706	0.93333	1.00000	0.93333	0.75000	0.71429	0.76923
	WOA-KELM	0.87851	0.07869	1.00000	0.86667	0.83333	0.85714	0.87500	0.92857	0.93333	0.92857	0.87500	0.68750
	SSA-KELM	0.86322	0.05392	0.88235	0.89474	0.90909	0.92857	0.76923	0.86667	0.82353	0.92308	0.77778	0.85714
	MsO-KELM	0.83379	0.11179	0.88889	0.93333	0.83333	0.88889	0.93333	0.83333	0.71429	0.56250	0.81250	0.93750
MCC	GWO-KELM	0.78620	0.08291	0.81168	0.86058	0.80917	0.69437	0.75094	0.80484	0.87082	0.59433	0.78954	0.87574
	HHO-KELM	0.77964	0.09857	0.79130	0.79524	0.65714	0.87082	0.75094	0.73207	0.58571	0.80917	0.87082	0.93318
	FPA-KELM	0.80578	0.10725	0.75523	0.86726	0.70588	0.65679	0.86190	1.00000	0.93333	0.79844	0.66696	0.81200
	WOA-KELM	0.79398	0.10018	0.93303	0.79524	0.72436	0.79427	0.87082	0.79524	0.79427	0.93303	0.58146	0.71807
	SSA-KELM	0.69917	0.11668	0.71569	0.86349	0.61301	0.93303	0.65052	0.58943	0.57353	0.73207	0.58146	0.73950
	MsO-KELM	0.82899	0.08910	0.78616	0.93333	0.80917	0.86726	0.93333	0.86349	0.80917	0.60467	0.81250	0.87083

**Table 9:** The prediction results on disease dataset Heart Disease.

Heart Disease													
Indicator	Algorithms	Mean	Std	1#	2#	3#	4#	5#	6#	7#	8#	9#	10#
ACC	GWO-KELM	0.69630	0.08733	0.66667	0.59259	0.81481	0.70370	0.70370	0.59259	0.66667	0.81481	0.81481	0.59259
	HHO-KELM	0.69259	0.08772	0.66667	0.59259	0.85185	0.66667	0.70370	0.59259	0.66667	0.81481	0.81481	0.59259
	FPA-KELM	0.70741	0.07115	0.70370	0.66667	0.77778	0.70370	0.74074	0.66667	0.66667	0.81481	0.77778	0.55556
	WOA-KELM	0.70370	0.09799	0.66667	0.62963	0.81481	0.74074	0.70370	0.59259	0.66667	0.77778	0.88889	0.55556
	SSA-KELM	0.66296	0.07305	0.59259	0.59259	0.77778	0.66667	0.66667	0.59259	0.62963	0.66667	0.81481	0.62963
	MsO-KELM	0.72222	0.07813	0.70370	0.70370	0.85185	0.77778	0.74074	0.66667	0.70370	0.81481	0.70370	0.55556
Sensitivity	GWO-KELM	0.57690	0.15682	0.60000	0.42857	0.90000	0.57143	0.57143	0.25000	0.63636	0.63636	0.63636	0.53846
	HHO-KELM	0.56976	0.15853	0.60000	0.42857	0.90000	0.50000	0.57143	0.25000	0.63636	0.63636	0.63636	0.53846
	FPA-KELM	0.63198	0.13636	0.70000	0.57143	0.80000	0.64286	0.64286	0.33333	0.54545	0.72727	0.81818	0.53846
	WOA-KELM	0.60937	0.16683	0.60000	0.50000	0.90000	0.64286	0.57143	0.25000	0.63636	0.63636	0.81818	0.53846
	SSA-KELM	0.42951	0.07305	0.40000	0.35714	0.70000	0.50000	0.50000	0.16667	0.54545	0.27273	0.54545	0.30769
	MsO-KELM	0.68853	0.11780	0.80000	0.64286	0.80000	0.78571	0.64286	0.41667	0.63636	0.72727	0.81818	0.61538
Specificity	GWO-KELM	0.80042	0.09743	0.70588	0.76923	0.76471	0.84615	0.84615	0.86667	0.68750	0.93750	0.93750	0.64286
	HHO-KELM	0.80042	0.09743	0.70588	0.76923	0.82353	0.84615	0.84615	0.86667	0.68750	0.93750	0.93750	0.64286
	FPA-KELM	0.77350	0.09368	0.70588	0.76923	0.76471	0.76923	0.84615	0.93333	0.75000	0.87500	0.75000	0.57143
	WOA-KELM	0.78702	0.10368	0.70588	0.76923	0.76471	0.84615	0.84615	0.86667	0.68750	0.87500	0.93750	0.57143
	SSA-KELM	0.85548	0.09534	0.70588	0.84615	0.82353	0.84615	0.84615	0.93333	0.68750	0.93750	1.00000	0.92857
	MsO-KELM	0.75307	0.12057	0.64706	0.76923	0.88235	0.76923	0.84615	0.86667	0.75000	0.87500	0.62500	0.50000
MCC	GWO-KELM	0.39037	0.17811	0.30062	0.20966	0.64242	0.43207	0.43207	0.14924	0.32024	0.61751	0.61751	0.18232
	HHO-KELM	0.38385	0.17766	0.30062	0.20966	0.70314	0.36690	0.43207	0.14924	0.32024	0.61751	0.61751	0.18232
	FPA-KELM	0.41245	0.14183	0.39445	0.34642	0.54880	0.41437	0.49728	0.34112	0.30062	0.61281	0.55874	0.10989
	WOA-KELM	0.40322	0.20024	0.30062	0.27857	0.64242	0.49728	0.43207	0.14924	0.32024	0.53300	0.76890	0.10989
	SSA-KELM	0.32280	0.15446	0.10847	0.23179	0.52353	0.36690	0.36690	0.15811	0.23295	0.29077	0.64466	0.30390
	MsO-KELM	0.44557	0.15081	0.43207	0.41437	0.68235	0.55495	0.49728	0.32127	0.38636	0.61281	0.43823	0.11602

**Table 10:** The prediction results on disease dataset Cleveland.

Cleveland													
Indicator	Algorithms	Mean	Std	1#	2#	3#	4#	5#	6#	7#	8#	9#	10#
ACC	GWO-KELM	0.67862	0.09806	0.70000	0.50000	0.76667	0.70000	0.73333	0.60000	0.76667	0.83333	0.60000	0.58621
	HHO-KELM	0.67862	0.09806	0.70000	0.50000	0.76667	0.70000	0.73333	0.60000	0.76667	0.83333	0.60000	0.58621
	FPA-KELM	0.68506	0.09372	0.60000	0.63333	0.70000	0.63333	0.86667	0.66667	0.76667	0.76667	0.70000	0.51724
	WOA-KELM	0.68839	0.09241	0.60000	0.63333	0.70000	0.66667	0.86667	0.66667	0.76667	0.76667	0.70000	0.51724
	SSA-KELM	0.64517	0.08481	0.60000	0.63333	0.60000	0.63333	0.83333	0.63333	0.7000	0.73333	0.53333	0.55172
	MsO-KELM	0.70184	0.08741	0.70000	0.60000	0.70000	0.70000	0.86667	0.66667	0.76667	0.80000	0.66667	0.55172
Sensitivity	GWO-KELM	0.67421	0.12466	0.72727	0.50000	0.92857	0.66667	0.69231	0.57143	0.78571	0.72727	0.64286	0.50000
	HHO-KELM	0.67421	0.12466	0.72727	0.50000	0.92857	0.66667	0.69231	0.57143	0.78571	0.72727	0.64286	0.50000
	FPA-KELM	0.59256	0.15431	0.45455	0.57143	0.71429	0.40000	0.69231	0.64286	0.85714	0.54545	0.71429	0.33333
	WOA-KELM	0.59923	0.14712	0.45455	0.57143	0.71429	0.46667	0.69231	0.64286	0.85714	0.54545	0.71429	0.33333
	SSA-KELM	0.45252	0.14078	0.27273	0.42857	0.50000	0.33333	0.61538	0.57143	0.71429	0.45455	0.35714	0.27778
	MsO-KELM	0.63519	0.11134	0.54545	0.57143	0.71429	0.53333	0.69231	0.64286	0.85714	0.63636	0.71429	0.44444
Specificity	GWO-KELM	0.68668	0.10728	0.68421	0.50000	0.62500	0.73333	0.76471	0.62500	0.75000	0.89474	0.56250	0.72727
	HHO-KELM	0.68668	0.10728	0.68421	0.50000	0.62500	0.73333	0.76471	0.62500	0.75000	0.89474	0.56250	0.72727
	FPA-KELM	0.77013	0.11023	0.68421	0.68750	0.68750	0.86667	1.00000	0.68750	0.68750	0.89474	0.68750	0.81818
	WOA-KELM	0.77013	0.11023	0.68421	0.68750	0.68750	0.86667	1.00000	0.68750	0.68750	0.89474	0.68750	0.81818
	SSA-KELM	0.81800	0.12426	0.78947	0.81250	0.68750	0.93333	1.00000	0.68750	0.68750	0.89474	0.68750	1.00000
	MsO-KELM	0.75906	0.11886	0.78947	0.62500	0.68750	0.86667	1.00000	0.68750	0.68750	0.89474	0.62500	0.72727
MCC	GWO-KELM	0.36245	0.19057	0.39747	0.00000	0.57309	0.40089	0.45701	0.19643	0.53452	0.63585	0.20536	0.22391
	HHO-KELM	0.36245	0.19057	0.39747	0.00000	0.57309	0.40089	0.45701	0.19643	0.53452	0.63585	0.20536	0.22391
	FPA-KELM	0.37742	0.17392	0.13876	0.26068	0.40089	0.30151	0.74863	0.33036	0.54833	0.47969	0.40089	0.16449
	WOA-KELM	0.38364	0.17219	0.13876	0.26068	0.40089	0.36370	0.74863	0.33036	0.54833	0.47969	0.40089	0.16449
	SSA-KELM	0.30108	0.17525	0.07087	0.26245	0.19094	0.33333	0.68958	0.26068	0.40089	0.39796	0.04725	0.35681
	MsO-KELM	0.40608	0.16562	0.34238	0.19643	0.40089	0.42426	0.74863	0.33036	0.54833	0.55849	0.33929	0.17172

**Table 11:** The prediction results on disease dataset Bupa.

Liver Disorders													
Indicator	Algorithms	Mean	Std	1#	2#	3#	4#	5#	6#	7#	8#	9#	10#
ACC	GWO-KELM	0.65571	0.12527	0.34286	0.74286	0.68571	0.71429	0.54286	0.62857	0.82857	0.68571	0.68571	0.70000
	HHO-KELM	0.67810	0.04583	0.65714	0.74286	0.74286	0.71429	0.71429	0.62857	0.65714	0.60000	0.65714	0.66667
	FPA-KELM	0.66429	0.10405	0.42857	0.74286	0.68571	0.71429	0.54286	0.65714	0.82857	0.65714	0.68571	0.70000
	WOA-KELM	0.68095	0.04349	0.65714	0.74286	0.74286	0.71429	0.71429	0.65714	0.65714	0.60000	0.65714	0.66667
	SSA-KELM	0.66714	0.07286	0.54286	0.77143	0.68571	0.71429	0.62857	0.65714	0.74286	0.54286	0.68571	0.70000
	MsO-KELM	0.70476	0.05216	0.68571	0.71429	0.77143	0.77143	0.65714	0.65714	0.77143	0.62857	0.65714	0.73333
Sensitivity	GWO-KELM	0.84923	0.09508	0.80000	0.72727	0.94444	0.72414	0.94118	0.90909	0.92308	0.94444	0.70370	0.87500
	HHO-KELM	0.80101	0.08283	0.80000	0.72727	0.83333	0.72414	1.00000	0.81818	0.76923	0.72222	0.74074	0.87500
	FPA-KELM	0.84368	0.09089	0.80000	0.72727	0.94444	0.72414	0.94118	0.90909	0.92308	0.88889	0.70370	0.87500
	WOA-KELM	0.81010	0.08898	0.80000	0.72727	0.83333	0.72414	1.00000	0.90909	0.76923	0.72222	0.74074	0.87500
	SSA-KELM	0.80546	0.10156	0.80000	0.75758	0.77778	0.72414	1.00000	0.90909	0.88462	0.61111	0.77778	0.81250
	MsO-KELM	0.82133	0.07947	0.80000	0.69697	0.88889	0.79310	0.94118	0.90909	0.84615	0.72222	0.74074	0.87500
Specificity	GWO-KELM	0.51041	0.21830	0.26667	1.00000	0.41176	0.66667	0.16667	0.50000	0.55556	0.41176	0.62500	0.50000
	HHO-KELM	0.55407	0.18497	0.63333	1.00000	0.64706	0.66667	0.44444	0.54167	0.33333	0.47059	0.37500	0.42857
	FPA-KELM	0.52458	0.20896	0.36667	1.00000	0.41176	0.66667	0.16667	0.54167	0.55556	0.41176	0.62500	0.50000
	WOA-KELM	0.55407	0.18497	0.63333	1.00000	0.64706	0.66667	0.44444	0.54167	0.33333	0.47059	0.37500	0.42857
	SSA-KELM	0.53247	0.19378	0.50000	1.00000	0.58824	0.66667	0.27778	0.54167	0.33333	0.47059	0.37500	0.57143
	MsO-KELM	0.59423	0.16648	0.66667	1.00000	0.64706	0.66667	0.38889	0.54167	0.55556	0.52941	0.37500	0.57143
MCC	GWO-KELM	0.33546	0.13057	0.05338	0.36364	0.42397	0.31030	0.16941	0.39304	0.52298	0.42397	0.28566	0.40825
	HHO-KELM	0.30891	0.13465	0.30641	0.36364	0.49010	0.31030	0.52899	0.33757	0.10256	0.19944	0.10758	0.34247
	FPA-KELM	0.33780	0.11549	0.12287	0.36364	0.42397	0.31030	0.16941	0.42714	0.52298	0.34381	0.28566	0.40825
	WOA-KELM	0.31786	0.13916	0.30641	0.36364	0.49010	0.31030	0.52899	0.42714	0.10256	0.19944	0.10758	0.34247
	SSA-KELM	0.29871	0.11377	0.21073	0.38925	0.37341	0.31030	0.39675	0.42714	0.25275	0.08251	0.14678	0.39747
	MsO-KELM	0.36706	0.11544	0.33333	0.34082	0.55437	0.38357	0.39286	0.42714	0.40171	0.25672	0.10758	0.47246

## Data Availability

The medical diagnostic datasets adopted in this paper are from the UCI machine learning repository (https://archive.ics.uci.edu/mL/index.php, accessed on 6 June 2022).
